# Purification, Identification, and Sensory Evaluation of Kokumi Peptides from *Agaricus bisporus* Mushroom

**DOI:** 10.3390/foods8020043

**Published:** 2019-01-29

**Authors:** Tao Feng, Yang Wu, Zhiwen Zhang, Shiqing Song, Haining Zhuang, Zhimin Xu, Linyun Yao, Min Sun

**Affiliations:** 1 School of Perfume and Aroma Technology, Shanghai Institute of Technology, No.100 Hai Quan Road, Shanghai 201418, China; fengtao@sit.edu.cn (T.F.); 13122386270@163.com (Y.W.); haley.zhang@reclassified.cn (Z.Z.); zxu@agcenter.lsu.edu (Z.X.); Lyyao@sit.edu.cn (L.Y.); sunmin@sit.edu.cn (M.S.); 2 Institute of Edible Fungi, Shanghai Academy of Agricultural Sciences, Key Laboratory of Edible Fungi Resources and Utilization (South), Ministry of Agriculture, National Engineering Research Center of Edible Fungi, 1000 Jinqi Road, Shanghai 201403, China; zhuanghaining@saas.sh.cn; 3 Department of Food Science, School of Nutrition and Food Sciences, Louisiana State University Agriculture Center, 111 Food Science Building, Baton Rouge, LA 70803, USA

**Keywords:** *Agaricus bisporus*, kokumi peptides, purification, sensory evaluation

## Abstract

*Agaricus bisporus* can enhance the umami and salty taste in chicken soup, and also has a high protein content, which indicates that there might be some kokumi taste compounds in this mushroom. Therefore, through ultrafiltration, gel permeation chromatography (GPC), and reverse phase-high performance liquid chromatography (RP-HPLC), some peptides in fresh *Agaricus bisporus* mushroom were isolated. Then, these peptides were identified by sensory evaluation and ultra performance liquid chromatography (UPLC) coupled quadruple time of flight mass spectrometry (Q-TOF-MS). The sensory evaluation results showed that the addition of aqueous extract isolated from *Agaricus bisporus* to model chicken broth did enhance chicken broth’s complexity, mouthfulness, and palatability. UPLC-Q-TOF-MS analysis found that Gly-Leu-Pro-Asp (Mw = 399.99) and Gly-His-Gly-Asp (Mw = 383.99) might act as key molecules to cause kokumi taste. In order to verify the kokumi taste of the above two peptides, they were synthesized by solid-phase synthesis and the taste properties of these two peptides were further characterized by descriptive sensory evaluation and taste intensity tests. This work indicated that it was feasible to produce kokumi peptides from *Agaricus bisporus*.

## 1. Introduction

Mushrooms often have good textures and flavors, and have been used as foods for many centuries. Because of their taste and nutritious value, mushrooms were widely used in the formulations of soups, stews, and sauces, as well as other savory dishes. In particular, the umami-like taste of mushrooms would impart palatability, complexity, and rich mouthfeel to culinary products [1].

To our best knowledge, sweet, sour, bitter, salt, and umami are the five fundamental tastes, contributing to the palatability of foods. However, kokumi is a sort of taste that differs from all five basic tastes, and is described by flavor characteristics such as mouthfulness, complexity, and continuity [2]. Kokumi taste substances have slight taste or even no taste by themselves, but they can enhance the flavor of the basic tastes, such as sweet, salty, and umami [3]. It was reported that various peptides, such as glutathione; γ-Glu-Val-Gly; and some γ-glutamyl di- and tri-peptides isolated from cheese, edible beans, soy sauce, and yeast extract, have kokumi tastes [4,5,6,7].

*Agaricus bisporus* is very popular with customers in many countries for its nutrients and special flavor. The flavor of *Agaricus bisporus* generally consists of volatile and nonvolatile compounds such as 1-octene-3-ol, mannitol, free amino acids, monosodium glutamate-like components, and 5’-nucleotides [8]. It has also been reported that fresh *Agaricus bisporus* or its dehydrated sample have antioxidative, antimicrobial, and hypocholesterolemic properties because of the peptides, free amino acids, 5’-nucleotides, and other compounds [9,10,11]. However, when added to a blank chicken broth, *Agaricus bisporus* can cause new taste sensations, such as mouthfulness and complexity, which have not been reported previously. 

Food stuffs with kokumi taste generally have high protein contents, and dried *Agaricus bisporus* contained 27% crude protein [8]. Therefore, the purpose of this paper was to isolate and identify the peptides associated with the kokumi taste and to evaluate their effects on some basic taste perception. This work may provide useful information to the deep processing of *Agaricus bisporus*.

## 2. Materials and Methods

### 2.1. Materials

Fresh *Agaricus bisporus* was cultivated in Guoshen Bio-Technique Co., Ltd. (Shanghai, China). Chicken broth was prepared using commercial chicken consommé seasoning from Unilever Co., Ltd. (Hefei, Anhui, China).

Food grade citric acid, sucrose, sodium chloride, monosodium glutamate (MSG), and caffeine were purchased from Sinopharm Chemical Reagent Co., Ltd. (Shanghai, China). L-glutathione was obtained from Sigma-Aldrich (St. Louis, MO, USA). Sephadex G-15 was acquired from Beijing Solarbio Science & Technology Co., Ltd. (Beijing, China). Methanol and formic acid were of high performance liquid chromatography (HPLC) grade and purchased from Sigma Aldrich (St. Louis, MO, USA).

### 2.2. Preparation of Water-Soluble Extract (WSE)

Fresh *Agaricus bisporus* was cleaned and the stalk was cut. The crude samples (1.2 kg) were sliced up and put in deionized water at a ratio of 1:1 (*w/w*). Processed samples were cooked for 120 min by autoclave (50YC16, SUPOR Electric Appliance Co., Ltd., Hangzhou, Zhejiang, China) at 40 kPa, 109.61 °C. Then, the samples were filtered with double gauze and the aqueous phase was obtained. The residue was re-extracted with water (1000 mL) at room temperature for 4 h. The aqueous phase was combined and centrifuged at 9338× *g*, 4 °C for 15 min. The supernatant (2.8 L) was concentrated to 0.6 L by a rotary vacuum evaporator (RE5210, Shanghai Ya Rong biochemical instrument factory, Shanghai, China) and lyophilized for 24 h by vacuum freeze dryer (LGJ-10, Beijing Bio Cool Experimental Instrument Co., Ltd., Beijing, China).

### 2.3. Ultrafiltration and Nanofiltration of the WSE 

In order to protect the membrane module from scratching, the WSE of *Agaricus bisporus* was dissolved in deionized water and pre-filtered with a 0.45 μm cellulose membrane. The pre-filtered solution was fractioned into two fractions using the ultrafiltration membrane (Shanghai Mosu Science Equipment Co., Ltd., Shanghai, China) HPS-3 (cut-off Mw = 3 kDa) under a pressure of 0.2 MPa. The fraction of molecular weight (Mw) < 3000 Da was processed by a nanofiltration membrane NF-1812 (cut-off Mw = 300 Da) under a pressure of 0.45 MPa. Ultrafiltration and nanofiltration were operated at room temperature (~25 °C). Finally, three fractions were achieved, namely, fraction U-I, Mw ≥ 3000 Da; fraction U-II, 300 Da ≤ Mw < 3000 Da; and fraction U-N-III, Mw < 300 Da. The three fractions were concentrated by a rotary vacuum evaporator, and then lyophilized for 24 h by vacuum freeze dryer (LGJ-10, Beijing Bio Cool Experimental Instrument Co., Ltd., Beijing, China). 

### 2.4. Amino Acid Analysis of Fraction U-I, U-II and U-N-III

#### 2.4.1. Free Amino Acid Analysis

An aliquot (1.0 g) lyophilized powder of the three fractions was hydrolyzed in 25 mL of 50 mg/mL trichloroacetic acid, respectively, and then filtered after 1 hour. Then, an aliquot (1 mL) filtrate was centrifuged at 9338× *g*, 10 min three times. The supernatant was pre-filtered with 0.22 μm membrane and 400 μL filtrate was placed in the liquid phase vial to determine free amino acids. 

#### 2.4.2. Total Amino Acid Analysis 

An aliquot (0.1 g) lyophilized powder of the three fractions was hydrolyzed in 8 ml of 6M HCL at 120 °C for 22 h, respectively. Then, 4.8 mL of 10M NaOH was added to the hydrolyzed sample for neutralization, and an aliquot (1 mL) solution was centrifuged at 9338× *g*, 10 min three times. The supernatant was pre-filtered with 0.22 μm membrane and 400 μL filtrate was placed in the liquid phase vial to determine total amino acids. 

### 2.5. Gel Permeation Chromatography (GPC) of Fraction U-II

According to sensory evaluation, an aliquot (1.25 g) lyophilized powder of fraction U-II was dissolved in 50 mL of ultra-pure water and pre-filtered with a 0.45 μm membrane. The sample was further separated by an automatic chromatographic separation device (Huxi Instrument Co., Ltd., Shanghai, China), equipped with a column (Φ2.6 × 60 cm) filled with a gel of Sephadex G-15. The elution was monitored at 220 nm and obtained at a flow rate of 0.75 mL/min using ultra-pure water as an eluent. Elution was collected every four minutes, and five fractions (F1–F5) were obtained. According to the elution profile, the individual fractions were combined and lyophilized. The powders of F1–F5 were stored at dessicator prior to use. 

### 2.6. Reverse Phase (RP)-HPLC Fractionation of F5 

According to the kokumi intensity perceived by sensory evaluation, Fraction F5 was selected and underwent further separation using an HPLC system coupled with a reverse-phase C18 column (250 × 10 mm, pursuit 5, Varian, Palo Alto, CA, USA). The mobile phase was monitored at 220 nm and consisted of 20% solvent A (acetonitrile) and 80% solvent B (water) at a flow rate of 3 mL/min. Total subfractions collected from F5 were S5.1–S5.4. All subfractions were liberated from solvent by lyophilization.

### 2.7. Separation and Identification of Kokumi Peptides in S5.1 by Ultra Performance Liquid Chromatography (UPLC)-Quadruple Time of Flight Mass Spectrometry (Q-TOF MS)

The identification of kokumi peptide in S5.1 was performed with a Waters Synapt Q-TOF MS (Milford, MA, USA) apparatus connected to an Acquity UPLC system, which was equipped with an automatic sampler (5 cm × 2.1 mm, 1.7 µm, Waters Acquity C18 column) and a binary solvent-delivery system, operating in electrospray ionization (ESI^+^) mode. The injection volume was 10 µL. The two kinds of mobile phases were elution A: Water/acetonitrile (*v/v*, 100/0.1) and elution B: Water/formic acid (*v/v*, 100/0.1). The program of gradient elution was performed as follows: 0–2 min: 0% A, 100% B; 2–3 min: 10% A, 90% B; 3–10 min: 100% A, 0% B. The flow rate was 0.3 mL/min. TOF-MS parameters were set as follows: Capillary voltage 3.2 Kvolts, cone voltage 20 Kvolts, source temperature 100 °C, desolvation temperature 400 °C. The dates were collected from *m*/*z* 20 Da to 1000 Da.

### 2.8. Solid Phase Synthesis of Target Peptides

The two target peptides were prepared by the solid phase synthesis method. The specific conditions were as follows: Venusil XBP C18 column (4.6 × 250 mm, 5 μm); injection volume: 20 μL, flow rate: 1.0 mL/min, detection wavelength: 220 nm. Two kinds of eluent were used as the mobile phase. Eluent A was 0.1% trifluoroacetic acid (TFA) in acetonitrile and eluent B was 0.1% TFA in water. The gradient elution conditions were as follows: 0–25 min: 12% A, 88% B; 25–30 min: 37% A, 63% B; 30 min–end: 100% B. 

Then, we identified the two synthetic peptides to make sure they were exactly the peptides we wanted using mass spectrometer (ZQ2000; Water). The specific detecting conditions were as follows: probe: ESI^+^; capillary voltage: 3.0 Kvolts; cone voltage: 50 Kvolts; extractor voltage: 5 Kvolts; desolvation temperature: 350 °C. If the MS spectra showed that the mass-to-charge ratio of the products was the same as that of our target peptides, then we could ensure that the synthetic peptides were successfully prepared.

### 2.9. Sensory Evaluation

#### 2.9.1. Training of Sensory Evaluation Panelists

The method of sensory evaluation was used according to Dunkel et al., with slight modifications [4]. The sensory evaluation panel was consisted of twenty members (ten men and ten women, aged 23–32 years), who had no experience of ageusia or taste disorders. Standard taste compounds dissolved in deionized water for training were used as follows: Citric acid (50 mmol/L, sour), sucrose (50 mmol/L, sweet), sodium chloride (25 mmol/L, salty), monosodium glutamate (3 mmol/L, umami), caffeine (1 mmol/L, bitter), and model chicken broth and the model chicken broth with 5 mmol/L reduced glutathione (kokumi). The panel was trained over two-week intervals for one year; therefore, everyone on this panel could sensitively recognize and distinguish different qualities of taste sensation. All panelists were trained in the separate sensory booths room at 20 ± 1 °C.

#### 2.9.2. Analysis of Taste Property

The lyophilized samples of WSE of *Agaricus bisporus*, ultrafiltration, and nanofiltration fractions, as well as GPC fractions and HPLC subfractions, were all dissolved in both deionized water and model chicken broth, respectively, and the pH value was adjusted to 6.5 with 0.2 M citric acid or 0.2 M sodium bicarbonate solution. These prepared solutions and model chicken broth were presented to the sensory evaluation panel, who were asked to describe the taste property or judge the intensity of kokumi (mouthfulness, complexity, continuity, etc.). The scale of the intensity was from 0 (not detected) to 5 (intensively perceived). 

#### 2.9.3. Sensory Evaluation of Synthetic Peptides and Their Corresponding Amino Acids Mixture

The method of sensory evaluation was used according to Frank et al., with slight modifications [12]. The target peptides were prepared as a series of solutions in model chicken broth with different concentrations (*w/w*); namely, 0.50%, 0.25%, 0.10%, 0.08%, and 0.05%. These prepared solutions and blank model chicken broth were presented to sensory evaluators, who were asked to describe the taste properties of the two peptides in chicken broth.

The taste characteristics of peptide and its mass-equivalent free amino acid mixture in water and chicken broth were analyzed and described. Then, we judged the intensity of sour, sweet, bitter, salty, umami, and kokumi taste, respectively. A five-point scale method was used in this study, that is, the scale of intensity was from 0 (no sensation) to 5 (intensively perceived).

### 2.10. Statistical Analysis

Sensory evaluation data were processed by one-way analysis of variance (ANOVA) to distinguish significant differences (*p* < 0.05) between various fractions and samples. 

## 3. Results and Discussion

### 3.1. Sensory Properties of WSE

Kokumi substances could contribute to the sensations of mouthfulness, complexity, and continuity when added to umami foods, while a solution of kokumi alone will not show these characteristics. Therefore, samples were evaluated in both distilled water and model chicken broth, respectively. The results of sensory evaluation indicated that WSE exhibited continuity, mouthfulness, complexity, and flavor-enhanced properties when added to a model chicken broth at a concentration of 1% (*w/w*).

Then, the WSE of *Agaricus bisporus* was separated by ultrafiltration and nanofiltration. The fraction U-II (300–3000 Da) imparted the kokumi taste when added to a model chicken broth at a concentration of 1% and displayed a stronger umami taste and slight bitter and astringent taste when added to water, while U-I had slight umami and bitter taste, and U-N-III had a salty and umami taste in water. Both failed to exhibit any kokumi taste in chicken broth under the same concentration (Table 1). On the basis of sensory evaluation, it can be considered that the fraction U-II can impart the kokumi sensation—continuity, mouthfulness, complexity, and flavor-enhanced effects.

### 3.2. The Amino Acid Contents Analysis of Fraction U-I, U-Ii, and U-N-Iii

The free amino acids (A) and total amino acids (B) of fraction U-I, U-II, and U-N-III were analyzed and amino acids in peptides (Table 2) were calculated (B minus A). From the analysis results, it could be seen that the amino acid content in peptide of fraction U-II (100.60 mg/g) was much higher than that of the other two (U-I: 53.57 mg/g; U-N-III: 48.67 mg/g), and its umami amino acids (50.31 mg/g) and sweet amino acids (24.6 mg/g) were the largest components relatively, which might be the key components that led to the kokumi taste of U-II in chicken broth.

As taste-active peptides referred to some low-molecular-weight oligopeptides, it was indicated that fraction U-II might contain some oligopeptides. Taste-active low-molecular-weight component played an important role in the aspect of enhancing flavors in various foods [13,14,15]. In addition, substances such as beans, yeast extract, shrimp paste, scallops, and soy sauce were reported to be sources of kokumi peptide, which also contained umami amino acids (Glu, Asp) and sweet amino acids (Gly, Ala). Therefore, it was speculated that the peptides in fraction U-II might have a strong sense of kokumi taste, so further separation of fraction U-II was done.

### 3.3. GPC Separation and Sensory Evaluation of Fraction U-II

**Figure 1:**
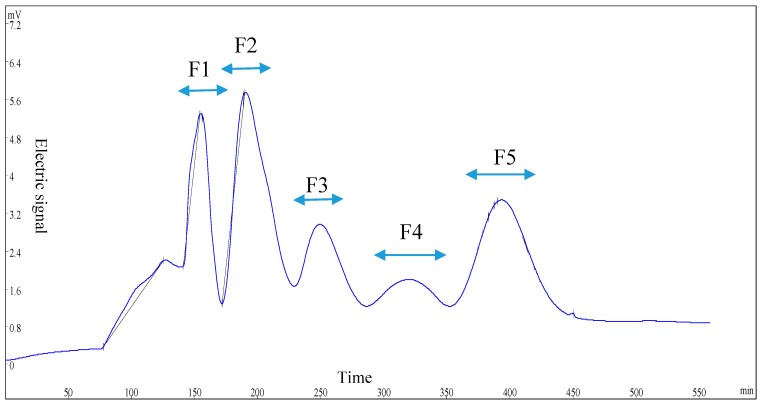
Gel permeation chromatography (GPC) chromatogram (λ = 220 nm) of fraction U-II.

**Figure 2:**
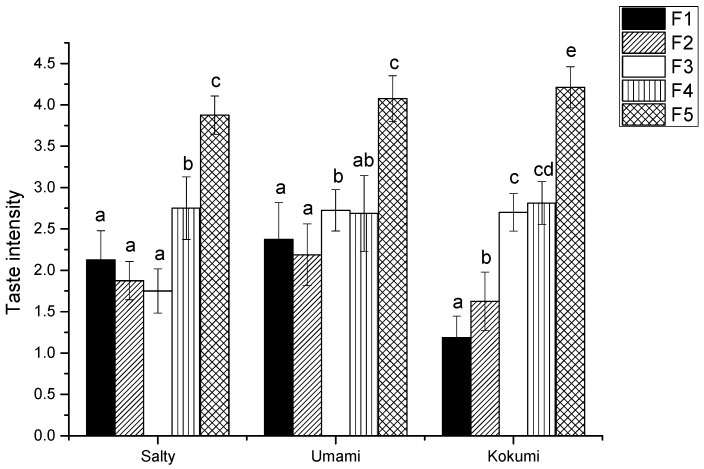
Salty, umami, and kokumi taste intensity of GPC fractions in model chicken broth. ^a, b, c, d, e^ The different letters in the same group represent that there are significant differences at the level of *p* < 0.05, and the same letters represent no significant difference.

Fraction U-II was separated by Sephadex G-15, and then five sub-fractions, F1, F2, F3, F4, and F5, were collected respectively, as shown in Figure 1. The five sub-fractions were also analyzed for their kokumi tastes in water and model chicken broth at the concentration of 1%. Interestingly, F5 exhibited the highest kokumi taste activity, such as mouthfulness, complexity, and continuity, in model chicken broth, and displayed significant differences (*p* < 0.05) in salty and umami solutions to the other four fractions (Figure 2). 

To further confirm the effect of kokumi taste of F5, the taste interaction between F5 and 1.0% sodium chloride or 0.5% MSG solution were investigated. One percent F5 was added to 1.0% sodium chloride and 0.5% MSG solution, respectively, so as to compare the changes of salty and umami intensity by sensory evaluation. The results showed that after F5 was added, salty intensity was improved from 1.9 to 2.6 and umami intensity was improved from 2.5 to 3.2, respectively, which was consistent with other results in the literature [3,16].

### 3.4. RP-HPLC Fractionation and Sensory Evaluation

**Figure 3:**
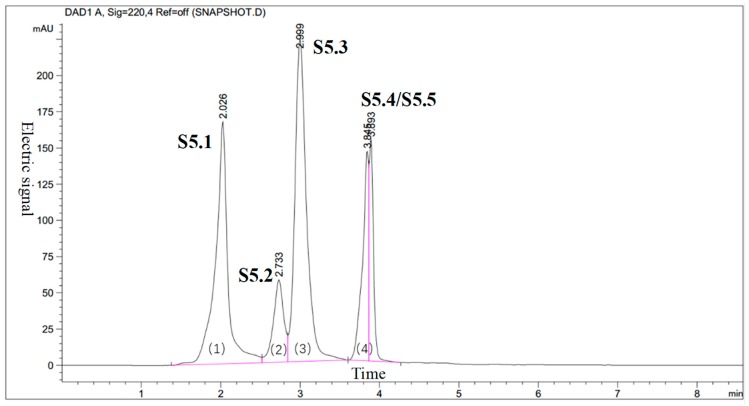
Reverse phase-high performance liquid chromatography (RP-HPLC) chromatogram of fraction F5 (five subfractions, S5.1–S5.5, were collected).

To decrease the complexity of the GPC isolated kokumi taste fractions, F5 was further separated by HPLC using a C-18 semi-preparative column. From this HPLC separation, five fractions were obtained (Figure 3), but S5.4 and S5.5 were not separated. The five fractions were analyzed for their kokumi tastes, and the fraction with the highest kokumi intensity was S5.1, according to the sensory evaluation.

### 3.5. Identification of Kokumi Peptides in S5.1

**Figure 4:**
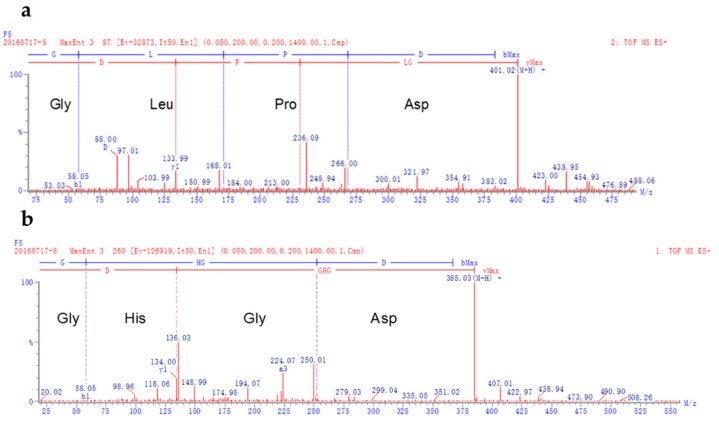
Liquid chromatography coupled quadruple time of flight mass spectrometry (LC-Q-TOF MS) spectrum (electrospray ionization (ESI^+^)) of the peptides Gly-Leu-Pro-Asp (**a**) and Gly-His-Gly-Asp (**b**) isolated from S5.1.

As the highest kokumi taste fraction, S5.1 was identified by UPLC-Q-TOF MS. The data revealed that the compounds with kokumi taste were recognized as Gly-Leu-Pro-Asp (Figure 4a) and Gly-His-Gly-Asp (Figure 4b). The flavors of peptides were influenced by the chain-length of peptides, and the type, sequence, and spatial structure of amino acids. Ohsu et al. studied the mechanism of kokumi, and indicated that some γ-Glu peptides, such as γ-Glu-Val-Gly, γ-Glu-Cys-Gly, and γ-Glu-Val, could elicit the kokumi taste by calcium-sensing receptor agonist [17]. Wang et al. reported that peptides with acidic amino acids such as Asp and Glu may elicit the kokumi taste in foods [18]. Both of the peptides obtained from this study contained Asp and Gly in the two terminals of the peptide chains. Some previous studies characterized kokumi taste peptides as having Asp, Gly, Glu, and Gln in their sequences [19,20,21], which was in agreement with the results of the present study. Dunkel et al. isolated various γ-Glutamyl peptides with kokumi taste from edible beans [4]. The absence of Glu in the peptides isolated in this study also resulted in kokumi taste. Similarly, a heptapeptide isolated from cultured puffer fish without Glu in its sequence also contributed to kokumi taste [22]. Therefore, the new peptides with Gly and Asp in their sequence were considered as contributors to the kokumi taste. 

### 3.6. Descriptive Sensory Evaluation of Synthetic Peptides

The two peptides were prepared by solid phase synthesis. The taste properties of the synthetic peptides in model chicken broth were described (Table 3). Both peptides showed kokumi taste characteristics such as mouthfulness, continuity, and complexity in chicken broth. Both peptides introduced umami and salty enhancement in chicken broth. In addition, synthetic peptides and the glutathione all showed a sour taste in chicken broth, which might be caused by residues of aspartic acid in the synthetic peptides and the presence of glutamate residues in glutathione standard. 

### 3.7. Sensory Evaluation of Synthetic Peptides and Their Corresponding Amino Acids Mixture

In order to further verify that the taste of peptides is not a taste superposition of their corresponding amino acids, the taste properties of synthetic peptides and their corresponding amino acid mixtures in water and chicken broth were compared with each other. 

Gly-His-Gly-Asp showed an astringent and bitter taste in water, while Gly, His, and Asp mixture showed umami, sweet, and sour tastes, respectively, in water. Gly-Leu-Pro-Asp exhibited an astringent and sour taste in water, while Gly, Leu, Pro, and Asp mixture exhibited umami, sweet, bitter, and sour tastes, respectively, in water. 

**Figure 5:**
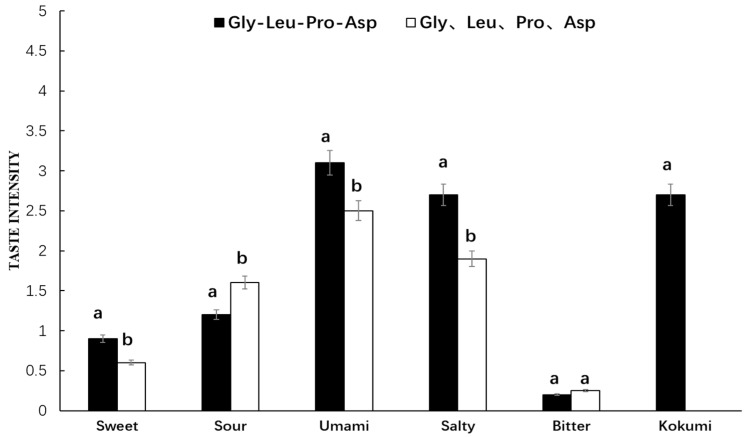
Sensory evaluation of peptide Gly-Leu-Pro-Asp and its mass-equivalent amino acids mixture in chicken broth. ^a, b^ The different letters in the same group represent that there are significant differences at the level of *p* < 0.05, and the same letters represent no significant difference.

As seen in Figure 5, Gly-Leu-Pro-Asp showed a strong umami, salty, and kokumi taste in chicken broth, while its amino acid mixture exhibited umami, salty, and sour tastes, but no kokumi taste, in chicken broth. The synthetic peptide and its amino acid mixture in chicken broth showed quite significant differences in umami, salty, sour, and sweet intensity, but their bitter intensity was relatively low with no significant difference. Thus, it was found that Gly-Leu-Pro-Asp in chicken broth with kokumi taste was quite different from that in water. However, the taste characteristics of its amino acid mixture in chicken broth were basically consistent with those in water.

**Figure 6:**
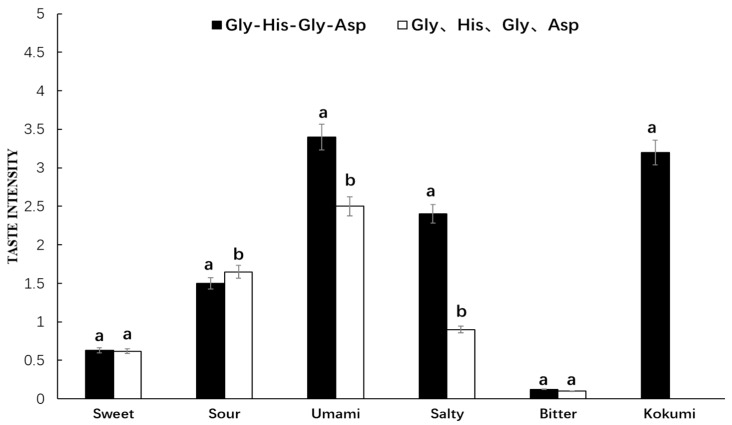
Sensory evaluation of peptide Gly-His-Pro-Asp and its mass-equivalent amino acids mixture in chicken broth. ^a, b^ The different letters in the same group represent that there are significant differences at the level of *p* < 0.05, and the same letters represent no significant difference.

It was seen from Figure 6 that Gly-His-Gly-Asp showed a strong umami, salty, sour, and kokumi taste in chicken broth, while its amino acid mixture exhibited umami and sour taste without showing kokumi taste in chicken broth. This synthetic peptide and its amino acid mixture in chicken broth showed dramatically significant differences in umami and salty intensity. Furthermore, their sour intensity was high, while their bitter intensity was low, and they both showed certain sweetness in chicken broth with no significant difference. It was thus indicated that Gly-His-Gly-Asp in chicken broth with obvious kokumi taste was quite different from that in water. The taste characteristics of amino acid mixture in chicken broth were basically consistent with those in water. 

In summary, the taste characteristics of the two peptides in water were different from their corresponding mass-equivalent amino acid mixture. The taste characteristics of the amino acid mixture showed a superposition of its individual amino acid taste, but the peptides in water showed their own different taste properties. The two peptides both exhibited a strong kokumi taste in chicken broth, and their amino acid mixtures only showed weak basic taste in chicken broth. 

## 4. Conclusions

(1) WSE of *Agaricus bisporus* was isolated and purified by ultrafiltration, nanofiltration, GPC Sephadex G-15, and RP-HPLC. S5.1, with the highest intensity of kokumi taste, was identified by UPLC-Q-TOF MS. Two peptides, Gly-Leu-Pro-Asp and Gly-His-Gly-Asp, in S5.1 were considered as important contributors to the kokumi taste, that is, mouthfulness, complexity, and continuity. 

(2) These two peptides were prepared by solid-phase synthesis. The kokumi taste of these two peptides was confirmed by descriptive sensory evaluation. The two kokumi peptides could increase salty and umami intensity when added to chicken broth. Therefore, we could apply them to condiment, adding less salt and MSG to achieve the same level of salty and umami effect. This application will cause people’s diets to become much healthier than before.

## Figures and Tables

**Table 1:** Sensory evaluation of water-soluble extract (WSE), ultrafiltration, and nanofiltration fractions in both water and model chicken broth.

Sample	Water	Model Chicken Broth
WSE	Umami, slight bitter	Mouthfulness, complexity
Fraction U-I	Slight umami, bitter	No kokumi sensation
Fraction U-II	Strong umami, bitter slight astringent	Richer, complexity, mouthfulness
Fraction U-N-III	Salty	No kokumi sensation

**Table 2:** Amino acids in peptides of different fractions from ultrafiltration.

Flavor	Amino Acids	U-I (mg/g)	U-II (mg/g)	U-N-III (mg/g)
Umami	Asp	6.00 ± 0.02 ^a^	15.00 ± 0.22 ^b^	7.90 ± 0.06 ^a^
	Glu	25.18 ± 0.34 ^a^	35.31 ± 0.07 ^b^	26.96 ± 0.22 ^a^
	Total	31.18 ± 0.03 ^a^	50.31 ± 0.11 ^b^	34.86 ± 0.13 ^a^
Sweet	Ala	2.08 ± 0.08 ^a^	3.58 ± 0.12 ^b^	0.74 ± 0.33 ^c^
	Gly	2.39 ± 0.07 ^a^	9.21 ± 0.01 ^b^	1.78 ± 0.12 ^c^
	Thr	3.25 ± 0.29 ^a^	8.66 ± 0.06 ^b^	3.36 ± 0.27 ^a^
	Ser	4.82 ± 0.01 ^a^	3.14 ± 0.02 ^b^	3.43 ± 0.09 ^b^
	Total	12.57 ± 0.36 ^a^	24.60 ± 0.04 ^b^	9.31 ± 0.01 ^c^
Bitter	Val	2.22 ± 0.11 ^a^	5.28 ± 0.31 ^b^	1.13 ± 0.07 ^c^
	Ile	2.78 ± 0.06 ^a^	3.56 ± 0.09 ^b^	0.89 ± 0.16 ^c^
	Leu	1.24 ± 0.15 ^a^	3.23 ± 0.01 ^b^	0.11 ± 0.04 ^c^
	His	0.78 ± 0.00 ^a^	1.62 ± 0.06 ^b^	2.05 ± 0.03 ^b^
	Phe	1.44 ± 0.04 ^a^	2.23 ± 0.12 ^b^	0.53 ± 0.20 ^b^
	Arg	0.22 ± 0.17 ^a^	2.16 ± 0.00 ^b^	0.19 ± 0.01 ^a^
	Met	0.03 ± 0.03 ^a^	1.13 ± 0.07 ^b^	0.29 ± 0.33 ^a^
	Total	7.97 ± 0.18 ^a^	19.21 ± 0.07 ^b^	4.26 ± 0.17 ^c^
Sweet and Bitter	Lys	1.08 ± 0.12 ^a^	4.39 ± 0.05 ^b^	0.85 ± 0.02 ^a^
	Pro	0.02 ± 0.06 ^a^	0.48 ± 0.13 ^b^	0.63 ± 0.05 ^b^
	Total	1.06 ± 0.02 ^a^	4.78 ± 0.02 ^b^	0.22 ± 0.01 ^c^
Flavorless	Cys	0.18 ± 0.00 ^a^	0.6 ± 0.04 ^b^	0.16 ± 0.04 ^a^
	Tyr	0.48 ± 0.04 ^a^	1.1 ± 0.03 ^b^	0.06 ± 0.44 ^c^
	Total	0.66 ± 0.04 ^a^	1.7 ± 0.15 ^b^	0.02 ± 0.01 ^c^
Total		53.57 ± 1.23 ^a^	100.60 ± 0.02 ^b^	48.67 ± 0.11 ^c^

^a, b, c^ The different letters in the same line represent that there are significant differences at the level of *p* < 0.05, and the same letters represent no significant difference.

**Table 3:** Sensory evaluation of peptides by solid phase synthesis in chicken broth.

Samples	Taste Properties
Blank chicken broth	Moderate umami; slight salty; light aftertaste; short lasting time
Blank chicken broth + Glutathione standard	Slight sour; enhanced umami and salty; a strong sense of mouthfulness and continuity
Blank chicken broth + Gly-His-Gly-Asp	Sour; a strong sense of umami; thickness and complexity; obvious punch on mouth; slightly enhanced salty
Blank chicken broth + Gly-Leu-Pro-Asp	Sour; overall taste similar to the standard; obvious kokumi taste
